# Phylogenetics of varied subtypes of avian influenza viruses in China: potential threat to humans

**DOI:** 10.1007/s13238-014-0036-1

**Published:** 2014-03-14

**Authors:** Weifeng Shi, Wei Li, Xianbin Li, Joel Haywood, Juncai Ma, George F. Gao, Di Liu

**Affiliations:** 1 School of Basic Medical Sciences, Taishan Medical College, Taian, 271016 China; 2 Network Information Center, Institute of Microbiology, Chinese Academy of Sciences, Beijing, 100101 China; 3 Shenzhen Institute of Advanced Technology, Chinese Academy of Science, Shenzhen, 518055 China; 4 University of Chinese Academy of Sciences, Beijing, 100049 China; 5 CAS Key Laboratory of Pathogenic Microbiology and Immunology, Institute of Microbiology, Chinese Academy of Sciences, Beijing, 100101 China; 6 Beijing Institutes of Life Science, Chinese Academy of Sciences, Beijing, 100101 China; 7 Office of Director-General, Chinese Center for Disease Control and Prevention (China CDC), Beijing, 102206 China

**Dear Editor,**

Since February 2013, infections with several novel avian-origin influenza viruses have occurred in Taiwan and the Mainland of China. The H7N9 influenza virus has caused 136 human infections with 44 deaths in the first influenza season, and has continued to cause infections during the current influenza season (Li et al., 2014). As of February 11th, 2014, a total of 331 human infections with 101 deaths in China have been reported (http://www.nhfpc.gov.cn). During the outbreak of H7N9 in the Mainland of China, Taiwan reported the first human infection of avian H6N1 influenza virus, which again hit the public (Shi et al., 2013a; Wei et al., 2013). In November 2013, the first infection of H10N8 influenza virus was reported by Nanchang Center for Disease Control and Prevention (Chen et al., 2014). Questions have since been raised as to how many novel avian influenza viruses could emerge and infect humans, and which have the potential to cause a pandemic?

The three novel avian-origin influenza viruses causing human infections were most likely transmitted from poultry to human (Chen et al., 2014; Chen et al., 2013; Shi et al., 2013a; Wu and Gao, 2013). Studies of the origin of these novel avian-origin influenza viruses have revealed that H7N9 and H10N8 have retained those surface protein-encoding genes that originated in wild birds during the transmission to domestic poultry (Chen et al., 2014; Cui et al., 2014; Liu et al., 2013). The internal genes of H7N9 and H10N8 were all derived from the H9N2 influenza viruses circulating within poultry (Cui et al., 2014). Multiple reassortment events within wild birds and poultry have resulted in the establishment of H7N9 and H10N8 viruses, respectively, and finally resulted in human infections. In contrast to H7N9 and H10N8, the H6N1 virus in Taiwan is probably the result of the reassortment of different lineages of poultry H6N1 viruses, though poultry H5N2 viruses might also been involved (Shi et al., 2013a).

The H7N9 virus in particular has diversified and experienced many dynamic reassortments within poultry (Cui et al., 2014; Li et al., 2013; Liu et al., 2013). The higher diversity of the internal genes makes the H7N9 virus rather different to the H5N1 and the pandemic H1N1 (09-pH1N1), whose surface protein-encoding genes exhibited more divergence during the first influenza season (Cui et al., 2014). There were at least 27 genotypes of the H7N9 virus discovered in the first influenza season, and novel genotypes continued to emerge in the 2013–2014 influenza season (Cui et al., 2014). The lack of an overwhelmingly dominant genotype of the H7N9 virus suggests that the virus has been continuing to evolve by means of rapid genetic reassortments, after they were transported to new regions, in order to adapt to local hosts. The genetic diversity has also endowed the H7N9 virus with functional diversity, in that some of the important amino acid sites: the receptor-binding site; the oseltamivir-resistance site; and mammalian host adaptation sites, have showed varied degrees of polymorphism (Li et al., 2013; Shi et al., 2013b; Wu et al., 2013).

The uncertainty of the combination of both the avian influenza virus subtypes and genotypes in poultry has made the preparedness for pandemic difficult. Therefore, tracing the potential evolutionary route of these avian influenza viruses in poultry is one of the pivotal steps for the control and prevention of novel human-infecting avian-origin influenza viruses. For this purpose, we performed the phylogenetic analyses of some of the prevalent avian influenza viruses in poultry in China. The results uncovered that H9N2 virus continued to act as genetic donor for the establishment of novel lineages of other subtypes of influenza virus, which could probably infect humans. The study also suggest that those hidden subtypes of influenza viruses, such as H1, H3, H4, and H11, which have not yet been found to infect humans, should not be neglected as hidden threats.

To provide insight into the avian influenza virus pool existing in poultry in China, we first searched the Influenza Virus Resources database maintained by NCBI for the avian influenza viruses (http://www.ncbi.nih.gov/genomes/FLU/). From 2006, there are a total of 2,146 HA gene sequences and 35 subtypes of avian influenza viruses (Supplementary Table 1). As shown in Fig. 1, the H9N2 virus in poultry has spread to nearly all provinces in China in recent years. As it is of a low pathogenicity to poultry, the transmission of this virus is likely to have been neglected through poultry transportations. The second most prevalent influenza virus in poultry is the H5 subtype, with the majority belonging to the H5N1 subtype and a few H5N2 viruses. The H5N1 virus has been isolated from at least 23 provinces in poultry, and has led to a policy of mass slaughter of infected poultry being enforced in China annually. It should be noted that though Qinghai Province has as yet had no reports of poultry infection with the H5/H9 subtype, the infection of H5N1 in wild birds shocked the world in 2005 and thereafter (Hu et al., 2011; Liu et al., 2005). As for the H7 subtype, most provinces with H7N9 human infection cases have isolated those viruses from poultry. The lack of adequate surveillance in some provinces may have resulted in those cases where human infection(s) have occurred but no virus sequence has been deposited in the database. Moreover, stable human-to-human H7N9 virus infection has not been confirmed yet and most patients had a history of exposure to poultry (Li et al., 2014).

**Figure 1:**
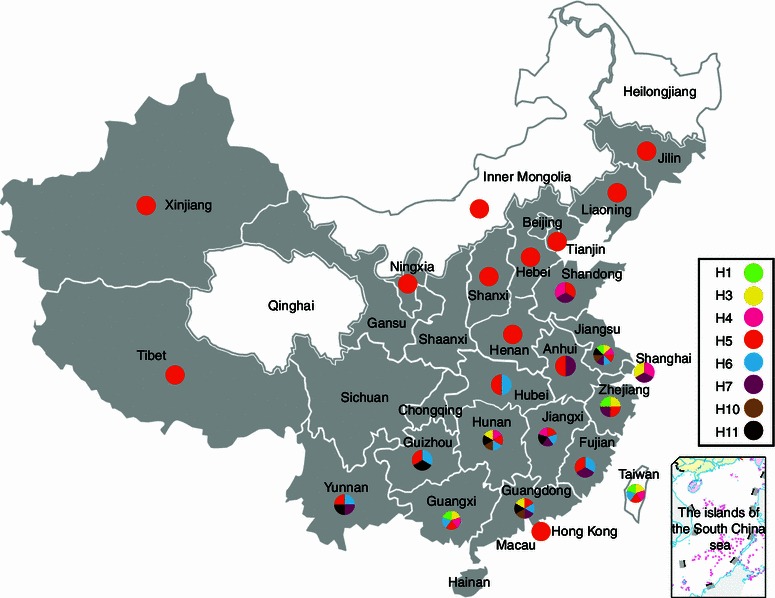
**Distribution of varied subtypes of influenza viruses within poultry of China (based on NCBI deposits)**. Different colors depict different HA subtypes, and the shaded regions show the emergence of H9N2 virus within poultry in that province

Three fatal infections with the H10N8 virus has been reported in Jiangxi Province, and this subtype has also been isolated from the live poultry market where the patient visited before onset of the illness (Chen et al., 2014). In addition, the H10 subtypes of influenza viruses have also been detected in Hunan (H10N3), wild birds of Dongting Lake and Guangdong (H10N8) ducks before.

The distribution also showed that H1 has been detected in four provinces, H3 in seven provinces, H4 in six provinces, H6 in ten provinces, and H11 in six provinces. These subtypes of viruses have been somewhat neglected for years, as there was no human infection reported in Mainland China. Interestingly, those provinces along the East shore of China have more than three subtypes detected, implying that migratory birds along the East Asian Flyway would have introduced varied influenza viruses into poultry. In Jiangxi and Hunan provinces, where Poyang Lake and Dongting Lake were located respectively, migratory birds stopped over each year for watering and breeding, and the subtypes of influenza viruses were also prosperous. It is interesting that the lakes and migratory birds might have caused Jiangxi and Hunan having varied influenza viruses in poultry, but Qinghai Lake has little effects to poultry in Qinghai Province. The reason might stand in that few domestic poultry feeding around Qinghai Lake but many domestic ducks were raised around Poyang Lake and Dongting Lake.

We subsequently performed phylogenetic analyses of the PB1 genes of the prevalent H5, H6, H7, and H10 subtypes of avian influenza viruses isolated in recent years in China (Fig. 2, see Supplementary Methods for details). From the phylogenetic tree, we observed that besides the H5 lineages evolving independently, there is also a lineage in which poultry H5 and H9N2 are co-circulating during 2007 to 2011 (Fig. 2A). The co-circulation of the two subtypes has also been reported in swine before (Shi et al., 2008). In particular, it was proposed that the H9N2 virus might have acted as “genetic donors” and contributed internal genes to the H5N1 virus (Guan et al., 1999). The H6 subtype virus has also been circulating in China in the past few years. It mostly infects ducks and has also evolved into multiple independent H6 lineages in Taiwan and the Mainland of China, respectively. The H6N1 virus causing human infection in Taiwan might be a reassortant strain of different Taiwan lineages. Although co-circulation of H6 and H9N2 has not been found in chicken in China, it was found in Muscovy ducks (Fig. 2B). For H7, it is well known that multiple lineages of the novel human-infecting avian-origin H7N9 influenza virus obtained their internal genes from H9N2 independently (Fig. 2C). In addition, a novel H7N7 lineage with H9N2-like internal genes was reported recently. In contrast, based on current surveillance data, only sporadic H10 infection cases have been reported and the H10 subtype has not yet established a stable lineage in China. Accordingly, the internal genes of the H10 virus strains from China were not clustered together in the tree (Fig. 2D). Alternatively, they were grouped together with different avian influenza virus lineages circulating in China. For instance, the human-infecting H10N8 obtained its internal genes from H9N2, while the internal genes of an H10N8 strain isolated from Guangdong in 2012 belong to a local lineage circulating in Guangdong in ducks and swine. Therefore, genetic reassortments between these subtypes lead to a huge genetic pool of avian influenza virus in which multiple subtypes and lineages are co-circulating.

**Figure 2:**
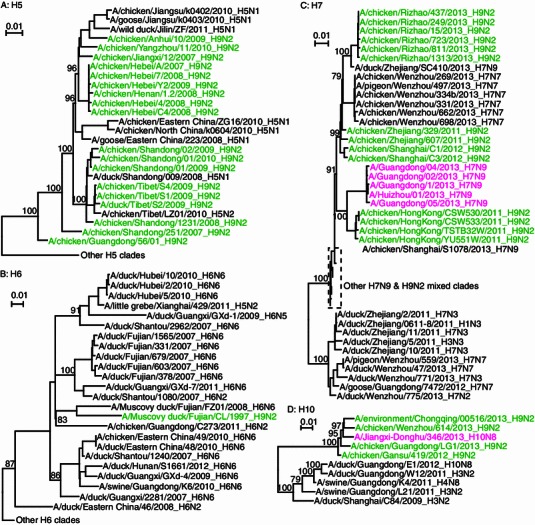
**Phylogenetic trees of PB1 gene of H5, H6, H7, and H10 subtypes of influenza viruses as examples for the other genes and other subtypes**. H9N2 viruses are in green and human infections are in magenta

It should be noted that the distribution map of poultry influenza virus was according to the sequence information deposited in GenBank. Surveillance has also shown that H9N2 were appeared in the Inner Mongolia and Heilongjiang province, though sequences have not been deposited (personal communications). Moreover, the lack of surveillance in most Northern provinces might omit the occurrence of influenza viruses other than H9N2 and N5 subtypes. The distribution reveals that the H9N2 and H5 viruses are prevalent within poultry in China. As H5 virus is still highly pathogenic to poultry and kills the majority of the infected chickens and some ducks, virus transmission ceases following the death of hosts. However, the H9N2 virus is of low pathogenicity to both chicken and ducks and transmits unnoticed, therefore the H9N2 virus has formed the background of the influenza virus infection in poultry in China. The ability of poultry to harbor many subtypes of influenza viruses combined with their habitats which are often shared with migratory birds carrying novel influenza viruses, creates a large opportunity for poultry to diversify influenza virus subtypes in circulation. As influenza virus possesses the nature to reassort with other subtypes, the background infection with the H9N2 virus in poultry (or simply as H9N2 carriers) would have unpredictable effects on the evolution of other subtypes.

Phylogenetics clearly reveals that there is a genetic pool of avian influenza virus in China. In the pool, subtypes of avian influenza viruses are able to evolve independently or through reassortments of gene fragments between and within the subtypes. This leads to the co-circulation of numerous influenza virus subtypes and lineages in poultry which, when combined with potential antigenic shift caused by genetic reassortments and point mutations of amino acids, provides a tough backdrop for the design and development of vaccines.

Currently, of the observed influenza viruses in poultry that have caused wide public concern over their potential to create a pandemic, H5N1, H7N9, and H10N8 have caused human deaths, and H6N1 and H9N2 have caused mild illness. The H1, H3, H4, and H11 subtypes on the other hand have not been found to infect humans to date and as yet cannot be predicted to whether or not they are able to overcome the host barriers to infect humans. However, we should not neglect these hidden threats, as we overlooked those for H7N9 and H10N8 in the past.

## Electronic supplementary material

Below is the link to the electronic supplementary material.

Supplementary material 1 (PDF 73 kb)
