# Supplementary material for: Phylogenetics of varied subtypes of avian influenza viruses in China: potential threat to humans
Source: Protein Cell. 2014 Mar 14;5(4):253–7. doi: 10.1007/s13238-014-0036-1 (PMC3978160; doi:10.1007/s13238-014-0036-1)
Supplement: Supplementary file 1 — Supplementary material 1 (PDF 73 kb) [file 13238_2014_36_MOESM1_ESM.pdf]

Supplementary

Materials and Methods

The PB1 gene sequences of each subtype were downloaded from The database at NCBI (Bao et al., 2008). We also limited the isolation region to “China”. For H5, H6, H7 and H9, we selected the sequences since the year 2008, 2007, 2005 and 2005, respectively. Then we performed Blastn using the four datasets against GenBank. In the Blast outputs, only sequences with subtypes different from that of the inputs (query datasets) were kept. Then the original four datasets and their own Blast output were combined, respectively. This gave four datasets, with each including 400, 496, 415 and 342 sequences. Multiple sequence alignment was performed using Muscle, respectively. Phylogenetic analysis was performed using RAxML (Stamatakis, 2014), with the GTRGAMMA model. One thousand bootstrap replicates were implemented. Full phylogenetic trees obtained in the first run were available on request. After the first run, we analyzed the phylogenetic trees and representative sequences were selected to compose smaller datasets for further phylogenetic analyses.

Reference

Bao, Y., Bolotov, P., Dernovoy, D., Kiryutin, B., Zaslavsky, L., Tatusova, T., Ostell, J., and Lipman, D. (2008). The influenza virus resource at the National Center for Biotechnology Information. Journal of virology 82, 596-601.

Stamatakis, A. (2014). RAxML version 8: a tool for phylogenetic analysis and post-analysis of large phylogenies. Bioinformatics.

Supplementary table 1, Distribution of poultry influenza virus subtypes in China.

Province	Host	Subtype
Anhui	Chicken	H5,H5N1,H9N2
	Duck	H5,H5N1,H7N9,H9N2
Beijing	Chicken	H9N2
	Duck	n/a
Chongqing	Chicken	H9N2
	Duck	H9N2
Fujian	Chicken	H5,H5N1,H9N2
	Duck	H5N1,H6N2,H6N6,H7N3,H7N7,H9N2
Gansu	Chicken	H9N2
	Duck	n/a
Guangdong	Chicken	H5,H5N1,H6N2,H7N9,H9N2
	Duck	H10N8,H11N9,H3N2,H5N5,H6N2,H6N6,H6N8,H9N2
Guangxi	Chicken	H1N2,H3N8,H5N1,H9N2
	Duck	H1N1,H1N2,H3N2,H3N8,H4N2,H5N1,H6N1,H6N2,H6N5,H6N6,H6N8
Guizhou	Chicken	H5N1,H9N2
	Duck	H11N9,H5N1,H6N1,H6N2,H6N5,H6N6,H6N8
Hainan	Chicken	H9N2
	Duck	n/a
Hebei	Chicken	H5N1,H5N2,H9N2
	Duck	H5N2
Henan	Chicken	H5N1,H9N2

	Duck	H5N1
Hong Kong	Chicken	H5N1, H9N2
	Duck	n/a
Hubei	Chicken	H5, H5N1, H9N2
	Duck	H5, H5N1, H5N5, H6N6, H9N2
Hunan	Chicken	H5, H5N1, H9N2
	Duck	H10N3, H11N9, H3N8, H4N2, H4N6, H4N9, H5N1, H5N2, H6N2, H6N6, H6N8, H6N9, H9N2
Jiangsu	Chicken	H10N9, H11N2, H5, H5N1, H5N2, H7N9, H9N2
	Duck	H11N3, H1N3, H3N2, H3N6, H4N2, H4N8, H5N2, H5N8, H6N5, H6N6, H9N2
Jiangxi	Chicken	H5, H5N1, H7N7, H7N9, H9N2
	Duck	H11N2, H11N9, H4N6, H4N9, H6N2, H7N3, H7N6, H7N7, H7N8, H7N9
Jilin	Chicken	H5, H5N1, H9N2
	Duck	H5, H9N2
Liaoning	Chicken	H5N1, H9N2
	Duck	H5, H5N1
Inner Mongolia	Chicken	H5
	Duck	n/a
Ningxia	Chicken	H5, H5N1, H9N2
	Duck	n/a
Shaanxi	Chicken	H9N2
	Duck	n/a
Shandong	Chicken	H5N1, H7N9, H9N2
	Duck	H4N6, H5N1
Shanghai	Chicken	H7N9, H9N2
	Duck	H3N2, H4N2, H4N6, H9N2

Shanxi	Chicken	H5N1, H9N2
	Duck	n/a
Sichuan	Chicken	H9N2
	Duck	n/a
Taiwan	Chicken	H4, H5N2, H6N1
	Duck	H1N3, H3N8, H4N2, H4N6, H4N8, H5N2
Tianjin	Chicken	H5, H9N2
	Duck	H5
Tibet	Chicken	H5, H5N1, H5N2, H9N2
	Duck	H9N2
Xinjiang	Chicken	H5, H5N1, H9N2
	Duck	n/a
Yunnan	Chicken	H5, H5N1, H9N2
	Duck	H11N9, H5N1, H6N2, H6N6, H7N6, H9N2
Zhejiang	Chicken	H7N7, H7N9, H9N2, H9N9
	Duck	H1N2, H1N3, H3N3, H5N1, H7N2, H7N3, H7N7, H7N9

n/a: not available
